# Social Value Orientation Moderated the Effect of Acute Stress on Individuals’ Prosocial Behaviors

**DOI:** 10.3389/fpsyg.2022.803184

**Published:** 2022-03-10

**Authors:** Liuhua Ying, Qin Yan, Xin Shen, Chengmian Zhang

**Affiliations:** Department of Psychology, Zhejiang Sci-Tech University, Hangzhou, China

**Keywords:** stress reactivity, Trier Social Stress Test, social value orientation, third-party punishment task, dictator game, tend-and-befriend

## Abstract

Acute stress is believed to lead to prosocial behaviors via a “tend-and-befriend” pattern of stress response. However, the results of the effect of acute stress on prosocial behavior are inconsistent. The current study explores the moderating effect of gender and social value orientation on the relationship between acute stress and individuals’ pure prosocial behaviors (i.e., pure prosociality and prosocial third-party punishment). Specifically, eighty-one participants were selected and underwent the Trier Social Stress Test (or were in the control group), followed by the third-party punishment task and the dictator game. The results showed that, in general, the main effect of condition or respiratory sinus arrhythmia (RSA) reactivity on individual prosocial behaviors was significant and did not vary between genders. Furthermore, social value orientation (i.e., prosocial or self-orientation) might moderate the impact of RSA reactivity on the amount of punishment in the third-party punishment task. That is, individuals with self-orientation exhibited more prosocial third-party punishment as RSA reactivity decreased, while the effect did not occur for individuals with prosocial orientation. Taken together, the findings of the current study provide further evidence for the “tend-and-befriend” hypothesis and highlight the underlying physical mechanisms as well as the individual dependence of the effect of psychosocial stress on individuals’ pure prosocial behaviors.

## Introduction

Stress is a common phenomenon in modern society. Exposure to stress changes individuals’ allostasis and leads to physiological responses that involve rapid and quick engagement of the sympathetic-adrenal-medullary (SAM) axis and a slow (peaking 21–40 min later) hypothalamic–pituitary–adrenal (HPA) axis (Dickerson and Kemeny, 2004). On the other hand, acute stress exposure also affects individual cognition and alters decision-making behaviors in social situations (Pechtel and Pizzagalli, 2011; von Dawans et al., 2021). Cannon (1932) posited the “fight-or-flight” hypothesis to characterize the physical and psychological responses to stress from an evolutionary perspective. That is, individuals tended to exhibit fewer prosocial behaviors and more fighting or flighting to the threat to enhance the likelihood of survival. However, researchers have recently questioned whether the “fight-or-flight” hypothesis could generalize to all social contexts. Therefore, Taylor and colleagues (2000) posited a “tend-and-befriend” theory in which stressed women have a desire to tend to children, affiliate with social groups and engage in prosocial behaviors to promote survival (von Dawans et al., 2019).

To date, accumulating evidence has shown that acute stress leads to a higher level of prosocial behaviors such as sharing, cooperation, trust, and altruistic behaviors (Vinker et al., 2013; Margittai et al., 2015; Singer et al., 2017; Potts et al., 2019; Zhang et al., 2019). For example, von Dawans et al. (2011) found that compared with participants under the control condition, both male and female participants engaged in substantially more prosocial behaviors (i.e., trust, trustworthiness, and sharing) after exposure to a social evaluative threat. However, the “tend-and-befriend” hypothesis on the effect of acute stress on prosocial behaviors is still far from conclusive. For example, Sollberger et al. (2016) found that although stress significantly increased their frequency of donation in male participants with low pro-environmental orientation, stress indeed reduced the amount of donation in all male participants. Potts et al. (2019) also revealed that acute stress might enhance or reduce the propensity to trust based on an individual’s unique pattern of physiological reactivity. These inconsistent results possibly resulted from the diversity of stress induction methods, the dependent variables employed to assess prosocial behaviors, the time interval between stress and measures of prosocial behavior, and the possible moderating variables (e.g., gender and personality).

Prosocial behavior refers to a range of positive behaviors, such as generosity, cooperation, and reciprocity, which are beneficial to other people, even at personal cost to the actor in some situations (Le et al., 2018). Those behaviors that benefit others but confer net costs to the person committing them are referred to as pure prosocial behaviors. Of those standardized experimental paradigms to study prosocial behavior, the dictator game is the most basic measure of pure prosociality, wherein a decision maker (i.e., the dictator) receives an endowment and splits endowment with an anonymous and completely powerless stranger (Engel, 2011). Meanwhile, third-party punishment is also considered a “pure” prosocial punishment to maintain fairness in human societies (Fehr and Gachter, 2002; Fehr and Fischbacher, 2004; Ginther et al., 2016). Conceptually, this idea refers to the circumstances in which people sacrifice their own resources to punish the norm violator without receiving any overt benefits, although they did not suffer from norm violation (Fehr and Fischbacher, 2004). Thus, it is helpful to extend the knowledge of the “tend-and-befriend” hypothesis by examining the relationships between acute stress and pure prosociality and punishment behaviors.

To date, several studies have examined pure prosociality and prosocial third-party punishment behaviors after individuals are exposed to acute stress. For example, Zhang et al. (2019) revealed that there was no significant effect of acute stress on generosity in the dictator game. However, another study examined the effect of acute stress on third-party intervention and found that acute stress can lead to more third-party helping behaviors but not more punishing behaviors (Zhen et al., 2021). That is, compared with participants in the control group, participants under acute stress tended to allocate more monetary units to the victim and exhibited more helping behavior in the scenario. In addition, Wang et al. (2020) study found that under a moderate but not serious level of unfairness, participants in the stress group tended to engage in more punishing behaviors and less helping behaviors than did participants in the control group, whereas under a high level of unfairness, there were no significant differences in punishing or helping behaviors between the stress and control groups.

One potential factor explaining the inconsistent results is gender. Nickels et al. (2017) examined individual competitive and cooperative tendencies under tasks (i.e., an Ultimatum Game, a Prisoner’s Dilemma, and a Prosocial Risk-Taking task) and found that relative to controls, male participants made more selfish decisions following exposure to acute stress, whereas female participants exhibited more other-oriented, generous, and cooperative behavior. Similarly, Zhang et al. (2019) revealed that there existed a significant gender-specific effect of stress-related cortisol change on prosocial behaviors. That is, men behaved more generously in the dictator game as stress-related cortisol reactivity increased, whereas a similar effect was not found in female participants. However, another study showed that the effects of acute stress on third-party intervention behaviors were not influenced by gender (Zhen et al., 2021). These mixed findings suggested that it is necessary to further consider the potential role of gender in individuals’ decision-making processes under acute stress.

Additionally, two recent meta-analyses suggested that individuals’ social decision-making following acute stress is also influenced by individuals’ attributes (von Dawans et al., 2021; Faber and Hausser, 2022). However, only Zhang et al. (2019) examined the moderating role of empathy concern in the effect of stress-related cortisol reactivity on subsequent prosocial decision-making (Zhang et al., 2019). Social value orientation is a feature of personality that reflects a stable individual difference in the way people weigh their own outcomes and compare them to others’ outcomes in social dilemmas (Messick and McClintock, 1968; Liebrand, 1984; van Lange, 1999) and is usually sorted into three categories: prosocial, individual, and competitor. Specifically, prosocial individuals attempt to equalize and/or maximize joint outcomes. Individualists are inclined to maximize their own welfare and provide less to others’ outcomes, and competitors attempt to maximize the relative difference between their own welfare and others’ outcomes (Liebrand, 1984; Van Lange and Kuhlman, 1994). Social value orientation was related to individuals’ decision-making behavior in social situations. For example, individuals with a prosocial orientation were more trusting than individuals with self-orientation (Derks et al., 2014). Similarly, Wei et al. (2016) showed that prosocial individuals might be less likely to be influenced by the selfish choices of group members and tend to make prosocial choices instead of proself behavior.

However, it is still not clear whether pure prosociality and prosocial third-party punishment under acute stress are affected by individuals’ social value orientation. Yu (2016) advanced a stress-induced deliberation-to-intuition (SIDI) model, which posits that in stressful situations, individuals may fall back on an automatic, intuitive, and habitual response and involve less slow, goal-oriented, deliberation reasoning. However, in the SIDI framework, it is inevitable that stress facilitates prosocial or antisocial behaviors in social situations. Individual innate responses are more likely context dependent and are influenced by their personality, including social value orientation (Steinbeis et al., 2015). Thus far, although there is a lack of direct examination of the effect of social value orientation on individuals’ pure prosociality and prosocial third-party punishment under stress, Haruno et al. (2014) showed that the presence of cognitive load made prosocial individuals behave more prosocially and individualists more individualistically, suggesting that the intuitive response from cognitive load is influenced by the behavioral pattern of prosocial individuals and individualists.

Therefore, to shed further light on the effect of acute stress on pure prosociality and prosocial third-party punishment, we designed an experiment where participants were exposed to a psychosocial stressor induced by the Trier Social Stress Test (TSST) or appropriate control condition individually and completed two game-theoretical social decision-making paradigms (i.e., R, the dictator game and the third-party punishment task). In addition, previous studies showed that the effect of acute stress on prosocial behaviors might also be influenced by the different temporal profiles of SAM versus HPA reactivity to stress (von Dawans et al., 2011; Potts et al., 2019). Heart rate is a valid and sensitive physiological measure to assess individuals’ rapidly occurring changes in physiological arousal (Narvaez Linares et al., 2020). Thus, individual differences in SAM stress responses were assessed via heart rate. Previous studies have suggested that RSA, an index of consecutive changes in heart beat that corresponds to changes in respiration, has been considered as an important physiological correlate of prosocial behavior (Kogan et al., 2014; Beffara et al., 2016; Zhang and Wang, 2019; Bello et al., 2020; Lazar and Eisenberger, 2022). Therefore, it was hypothesized that the condition or RSA reactivity of acute stress would increase generosity in dictator games and the frequency and amount of punishment in the third-party punishment task. Moreover, the effects of condition or RSA reactivity on prosocial behaviors (i.e., generosity in dictator games and the frequency and amount of third-party punishment) were moderated by gender, with more prosocial behaviors in female participants than males and social value orientation, with more prosocial behaviors in participants with prosocial but not proself orientation.

## Materials and Methods

### Participants

Eighty-one participants were recruited to complete the current study through posted fliers and an existing online recruitment pool (33 males, 48 females; mean age = 19.55 years older, *SD*_age_ = 1.47). All participants were randomly assigned to be in either the stress condition (*n* = 49) or the control condition (*n* = 32). Exclusion criteria included a history of medical or psychiatric illness and alcohol/caffeine use on scheduled testing days. Additionally, students of psychology or economics who were familiar with the experimental procedure were also excluded from the current study. Written informed consent was obtained from all participants after giving a detailed explanation of the experiment. The study was approved by the Ethics Committee of Zhejiang Sci-Tech University. Participants were told that they would receive monetary compensation based on their task performance.

### Procedure

**FIGURE 1:**
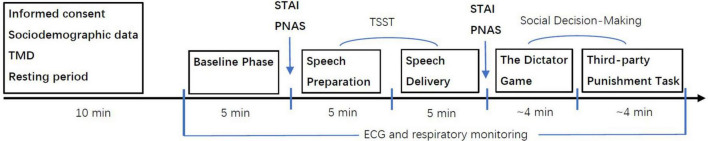
Flow chart of the experiment procedure. TMD, triple dominance measure; STAI, State-Trait Anxiety Inventory; PANAS, positive and negative affect schedule; TSST, Trier Social Stress Test.

Upon arrival at the laboratory, participants received a detailed account of the procedure of the experiment and were then provided with written informed consent to read and sign (see Figure 1). Then, they sat in a room with computers that lab assistants attached ECG sensors, a respiratory belt, and CNAP 500 devices to them. During a resting period of 5 min, all participants completed a series of questionnaires that were used to measure individuals’ social value orientation and other sociodemographic variables. During the 5-min baseline period followed by a 10-min laboratory acclimation period, the participants were asked to rest quietly and relaxedly and viewed a neutral picture presented on the monitor. At the end of the baseline period, the participants were asked to rate their current affect using the State-Trait Anxiety Inventory (STAI) and the Positive and Negative Affect Schedule (PANAS).

Then, two research associates entered the experiment room and explained the task as follows: “Now, you have an interview for finding a job. After asking for 5 min to prepare, you will make a 5-min presentation to state why you are qualified for this job. Your performances will be videotaped and evaluated by the researcher for overall content, clarity, and delivery.” Participants under the control condition were asked to sit quietly in the phase of prepare and public talking. Immediately after the interview, the two research associates left the room, and participants completed the STAI and the PANAS. Subsequently, after receiving a short instruction, the participants continued to complete two randomly presented social decision-making tasks (i.e., the dictator game and the third-party punishment task). Finally, the participants sat and relaxed for 5 min, after which they received payment for their participation.

### Social Decision-Making Paradigms

#### The Dictator Game

The modified version of the dictator game (Fehr and Fischbacher, 2004; Henrich et al., 2006) consisted of a “dictator” who is endowed with 100 tokens and is asked to allocate any portion of these tokens (or nothing at all) to a “receiver,” who has no choice but accepts a proposed allocation. At the beginning of the task, participants were assigned to the allocator role, although they were told that the role was randomly decided by computer programming. In eight trials, the dictator decided how to allocate the 100 tokens. To improve the authenticity of the task, the receivers’ names were presented on the left screen of the computer. The amount of tokens dictator allocated from the receivers was used to evaluate the level of participants’ generosity in the dictator game.

#### Third-Party Punishment Task

The third-party punishment task is based on the dictator game and adapted from a previous study (Leliveld et al., 2012). In the third-party punishment task, each participant (Player C) was invited to play with two other players (Player A: the dictator, and Player B: the victim) in an anonymous online economic game. Specifically, the participant was told that Player A (the proposer) was endowed with 100 coins and has a right to determine how to allocate the endowment to Player B (a receiver), who only accepted the allocation from the proposer. Each participant (Player C), as a third-party observer endowed with 50 coins, decided whether and how much coins he paid to punish the proposer (Player A) based on the proposer’s distribution. Throughout the experiment, these participants were led to believe that they were playing with two other actual participants who in fact were computer generated.

In each trial, Players A and B were endowed with 100 coins. Player A allocated one amount (49, 46, 43, 40, 33, 30, 26, 23, 15, 12, 8, 5) to the recipient. After observing the unfair allocations, the participants (Player C) needed to decide whether to punish the allocator, and if so, how much to pay for the punishment—up to a total of fifty of their own coins. Player C was able to punish player A at a 3:1 rate. For instance, if participants paid for 10 coins to punishment, the allocator lost 30 coins. Finally, they were told that the rest of the endowment in the selected random trial was transferred to the participants’ reward at the end of the experiment.

### Tier Social Stress Test

Acute stress was induced using the adapted TSST (Kirschbaum et al., 1993), which is widely used to experimentally study the stress response in human subjects. The effectiveness of TSST in inducing individual stress responses in Chinese culture has been evidenced by these previous studies (Yang et al., 2011). In addition, previous studies found that although the TSST could induce moderate stress by combining several stressful components, it is also difficult to disentangle the specific role of different aspects of the stressor (Allen et al., 2016). Furthermore, previous studies have shown that only public speaking can provoke significant subjective and physiological responses (Garcia-Leal et al., 2014; Narvaez Linares et al., 2020). Thus, in the current study, a public speaking task of the TSST was used to induce acute psychosocial stress. Specifically, after establishing a clear baseline, participants were first instructed to prepare an upcoming fictitious job interview for 5 min and asked to give a speech in front of the two research associates for 5 min. The two associates showed a neutral facial expression during the tasks. The participants were told that their speech would be videotaped and that the tape would later be evaluated by the researcher for overall content, clarity, and delivery. During the speech task, if the participant stopped talking (approximately 10 s), he or she would be asked to continue speaking, with the researcher saying, “Please continue. I will tell you when your time is up.” In addition, previous study found that there existed the modest activation of the sympathetic nervous system (SNS) among those participants under the placebo-TSST (Het et al., 2009). Thus, we chose a pure resting control condition to preclude an increase of SNS system from the placebo-TSST from the effect of RSA reactivity on prosocial behaviors in current study.

### Social Value Orientation

Social value orientation was measured by the triple dominance measure (TDM; Van Lange et al., 1997). Participants were asked to make nine choices among combinations of outcomes for oneself and for another person. The participants were then asked to choose the option they preferred the most each time. They were asked to imagine that the points were valuable to them and that how much they would obtain depended on their choice as well as on the choice independently made by the other person. Across the nine choices, the number of points in each cell was varied in steps of 10 points. The choices were presented in a random order with the positions of the different alternatives (left, middle, or right) counterbalanced.

### Measures of Psychological and Physiological Responses

#### Psychological Stress Responses

Participants’ anxiety was assessed with the 6-item short-form of the state-trait anxiety inventory (STAI; Spielberger et al., 1970; Marteau and Bekker, 1992). An example item is “I feel anxious when I speak in front of a group.” Participants responded to each item on a 4-point Likert scale ranging from “not at all like me” to “a lot like me.” Higher scores reflected greater levels of social anxiety. The short version of the STAI has been found to have good psychometric properties (Marteau and Bekker, 1992). In the current study, Cronbach’s alpha of the scale was 0.77 during the baseline period and 0.91 poststress.

Additionally, the PANAS (Watson et al., 1988) was used to assess participants’ affect state shortly before and immediately after the stress task. Participants responded to each item of 10 items for positive affect and 10 items for negative affect on a 5-point Likert-scale ranging from “not at all” to “extremely.” Higher scores reflected greater levels of positive and negative affect. The PANAS has been found to have good psychometric properties (Watson et al., 1988). In the current study, Cronbach’s alpha of the scale was 0.89 during the baseline period and 0.87 at poststress.

#### Physiological Measurements

Electrocardiograms (ECGs) were recorded with a Biopac ECG100 amplifier by using the standard lead II configuration. The sampling rate for ECG signals was set at 1,000 Hz and filtered using a bandpass of 0.5–35 Hz. A respiration signal was measured with a Biopac RSP100C amplifier with a bandpass filter of 0.5 and 1 Hz and was recorded using TSD201 transducers embedded in a respiratory belt around the participants’ chest (at the level of the fifth thoracic vertebrae). The ECG and respiration data were recorded and integrated by Biopac MP150 hardware and AcqKnowledge software packages (Biopac MP150, AcqKnowledge; Biopac System, Inc., Goleta, CA, United States).

R waves in the ECG signal were automatically identified and manually checked for missing or mislabeled R waves. Interbeat intervals (IBIs) were calculated as the interval (in milliseconds) between successive R waves in the electrocardiogram. RSA was estimated based on the ECG and respiration data by an AcqKnowledge automated function, which followed the “peak-to-valley” method (Berntson et al., 1997). The RSA during three experimental periods (i.e., baseline RSA, stress RSA, and poststress RSA) was calculated using the average 5 min RSA for each period. RSA reactivity was calculated by subtracting the average stress RSA from the average baseline RSA. RSA rebound was calculated by subtracting the average poststress RSA from the average baseline RSA.

### Statistical Analysis

All statistics were analyzed using SPSS version 19 (SPSS, Inc., Chicago, IL, United States). Independent-sample *T* tests or chi-square tests were performed to detect the differences in age, gender, and trait anxiety between the stress and control conditions.

Respiratory sinus arrhythmia levels and subjective stress were analyzed using repeated-measures ANOVAs with the between-subjects factor condition (stress, control) and the repeated factor phase (three for RSA level, two for status anxiety, and two for negative/positive emotion). The Greenhouse–Geisser correction was used when the requirement of sphericity was violated. Simple effects tests using the MANOVA command in SPSS were conducted to examine the potential differences in RSA level and subjective stress at a single phase between the stress condition and the control group.

Then, the main effects of the condition on prosocial behaviors (i.e., the frequency and amount of punishment and the denoted amount in the dictator game) were analyzed using three ANOVA. Because RSA reactivity was a continuous variable, linear regression analyses were used to determine its main effect on these prosocial behaviors. Finally, ANOVAs of the interaction effect of condition and gender or social value orientation on prosocial behaviors were performed. Hierarchical linear regressions (HLRs) were used to examine the moderating effect of social value orientation or gender on the effect of RSA reactivity on prosocial behaviors. In these analyses, the independent variable (i.e., RSA reactivity) and the moderating variables (gender or social value orientation) were centered on their respective means to reduce the multicollinearity between main effects and the interaction term and to increase the interpretability of the weights for interaction terms (Cohen and Cohen, 1983). Specifically, the independent variable and the moderating variables were entered into the regression model in Step 1. Then, the interaction between the independent variable and the moderating variable was entered in Step 2. If the interaction effects were significant, the simple slope tests were further conducted using the simple slopes syntax (Schubert and Jacoby, 2004) to tested whether the simple slopes of the interaction were significantly different from zero.

## Results

### Demographic Variables and Preliminary Analyses

The stress groups and the control groups did not significantly differ in age [*t*_(87)_ = −0.47, *p* > 0.05], gender (chi-square = 3.48, *df* = 1, *p* > 0.05), or trait anxiety [*t*_(87)_ = −1.36, *p* > 0.05, see Table 1].

**TABLE 1:** Characteristics of physical and psychological stress responses (*n* = 81).

Variables	Stress condition (*M* ± *SD*)	Control condition (*M* ± *SD*)
Age	19.506	1.501
RSA 1	4.088	0.401
RSA 2	4.027	0.422
RSA 3	4.068	0.381
Status anxiety 1	10.469	3.038
Status anxiety 3	13.567	5.123
Positive emotion 1	26.370	7.383
Positive emotion 3	25.593	8.330
Negative emotion 1	14.889	5.327
Negative emotion 3	18.196	8.288

*1, “at baseline phase”; 2, “during the TSST”; 3, “post TSST.”*

### Manipulation Check

To test the efficacy of the stress manipulation, several repeated-measures ANOVAs were conducted with the between-factor condition (stress, control) and the repeated-measure factor phase (repeated factor: three for RSA level, two for status anxiety, and two for negative/positive emotion). The results showed that there was a significant interaction between the condition and phase [RSA level at baseline, stress phase, postexposure; *F*_(1.67,131.78)_ = 6.40, *p* < 0.01, partial η^2^= 0.08]. Simple effects tests indicated that in the stress condition, the RSA level significantly decreased after exposure to the TSST and was restored to the baseline level after the TSST (*p* < 0.001). The RSA level during the stress period was significantly lower than the baseline level of RSA (*p* < 0.001). Regarding the control condition, compared with the levels of RSA during the baseline phase and the restoration phase, the level of RSA was not significantly different during exposure to the TSST (*p* = 0.41). Moreover, when the RSA level was in the control condition, the level of RSA was significantly lower in the stress condition than in the control condition (*p* < 0.05).

The analysis of status anxiety showed that there was a significant interaction between condition and phase [status anxiety at baseline and posttask; *F*_(1,79)_ = 38.15, *p* < 0.001, partial η^2^= 0.33]. Simple effects tests revealed that there was a significant difference in status anxiety between the baseline and posttask in the stress condition [*F*_(1,79)_ = 82.66, *p* < 0.001] but not in the control condition [*F*_(1,79)_ = 0.35, *p* = 0.55]. The level of status anxiety was significantly higher posttask than at baseline (*p* < 0.001).

In addition, these analyses of the positive and negative emotions showed that while the interaction between group and phase (negative emotion at baseline and posttask) was significant [*F*_(1,79)_ = 29.24, *p* < 0.001, partial η^2^= 0.27], the interaction between group and phase (positive emotion at baseline and posttask) was found to be non-significant [*F*_(1,79)_ = 1.92, *p* = 0.17, partial η^2^= 0.02]. Simple effects tests revealed that a significant difference in negative emotion existed in the stress condition [*F*_(1,79)_ = 47.15, *p* < 0.001] but not in the control condition [*F*_(1,79)_ = 1.97, *p* = 0.17]. Moreover, the level of negative emotion was significantly higher posttask than at baseline (*p* < 0.001).

### Main Effects of Acute Stresses on Prosocial Behaviors

To examine whether acute stresses might influence prosocial behaviors (i.e., pure prosociality and prosocial third-party punishment), two one-way ANOVAs were performed. The results revealed that there was a significant main effect of condition on the amount of punishment in the third-party punishment task [*F*_(1,79)_ = 5.34, *p* = 0.02, partial η^2^= 0.06] and the amount invested in the dictator game [*F*_(1,79)_ = 4.07, *p* = 0.047, partial η^2^= 0.05], but the effect of condition on the frequency of third-party punishment was not significant. After exposure to acute stress, third-party participants were inclined to allocate more tokens to receivers as victims (*M* = 7.98, *SD* = 6.54, *p* = 0.02) and donated more tokens to receivers (*M* = 37.46, *SD* = 16.84, *p* = 0.047) in the dictator game than their counterparts in the control condition did (*M*_*in third*−*party punishment task*_ = 4.96, *SD* = 4.21; *M*_*indictator game*_ = 29.41, *SD* = 18.62).

In addition, two linear regression analyses were used to examine the effects of RSA reactivity on the frequency and amount of punishment behaviors in the third-party punishment task and generosity in the dictator game. The results revealed that there were significant negative effects of RSA reactivity on the frequency (β = −0.33, *t* = −3.10, *p* < 0.01) and amount (β = −0.40, *t* = −3.85, *p* < 0.001) of punishment in the third-party task and the donated amount in the dictator game (β = −0.40, *t* = −3.89, *p* < 0.001).

### Moderating Effects of Gender and Social Value Orientation

First, three-way ANOVAs were used to determine the interaction effect between three independent variables (i.e., condition, gender, and social value orientation) on prosocial behaviors (i.e., pure prosociality and prosocial third-party punishment). The results showed that there was no significant interaction effect between condition and gender on the frequency [*F*_(1,68)_ = 0.58, *p* = 0.45, partial η^2^= 0.01] and amount [*F*_(1,68)_ = 0.03, *p* = 0.86, partial η^2^= 0.000] of punishment in the third-party task or the donated amount in the dictator game [*F*_(1,68)_ = 0.03, *p* = 0.86, partial η^2^= 0.00]. Moreover, there was no significant interaction effect of condition and social value orientation on the frequency [*F*_(1,68)_ = 0.66, *p* = 0.42, partial η^2^= 0.01] and amount [*F*_(1,68)_ = 0.45, *p* = 0.51, partial η^2^= 0.01] of punishment on the third-party task or on the donated amount in the dictator game [*F*_(1,68)_ = 0.07, *p* = 0.80, partial η^2^= 0.000]. In addition, the results revealed that there was no significant three-way interaction effect with condition, gender, and social value orientation on the frequency [*F*_(1,68)_ = 0.23, *p* = 0.63, partial η^2^= 0.00] and amount [*F*_(1,68)_ = 0.00, *p* = 0.97, partial η^2^= 0.000] of punishment in the third-party task or the donated amount in the dictator game [*F*_(1,68)_ = 0.32, *p* = 0.57, partial η^2^= 0.01].

**FIGURE 2:**
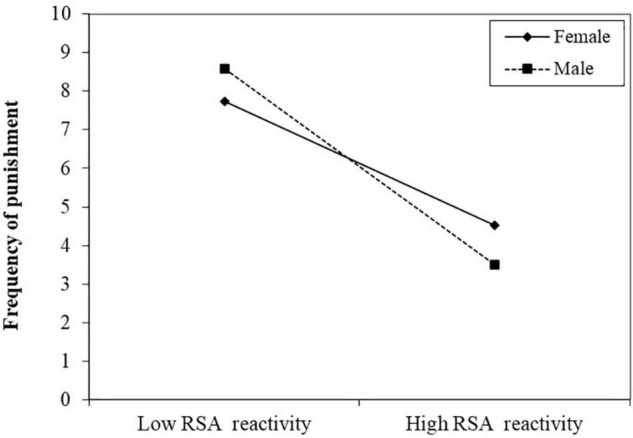
Simple slope plots of RSA reactivity × gender on the frequency of punishment in third-party punishment task.

Then, linear regression analyses examined the interaction effect between RSA reactivity and gender on prosocial behaviors (i.e., pure prosociality and prosocial third-party punishment). The results did not reveal significant interaction effects of RSA reactivity and gender on the amount of punishment behavior (β = −0.13, *t* = −0.99, *p* > 0.05) or on the donated amount in the dictator game (β = −0.09, *t* = −0.71, *p* < 0.05). However, significant interaction effects of RSA reactivity and gender on the frequency of punishment behaviors were detected (Δ*R*^2^ = 0.06, β = −0.29, *t* = −2.26, *p* < 0.05). Simple slope analyses showed that while for female participants, RSA reactivity did not influence the frequency of punishment (β = −0.53, *t* = −3.44, *p* < 0.01; Figure 2), male participants exhibited a lower frequency of punishment as RSA reactivity increased (β = −0.25, *t* = −1.72, *p* > 0.05; Figure 2).

**FIGURE 3:**
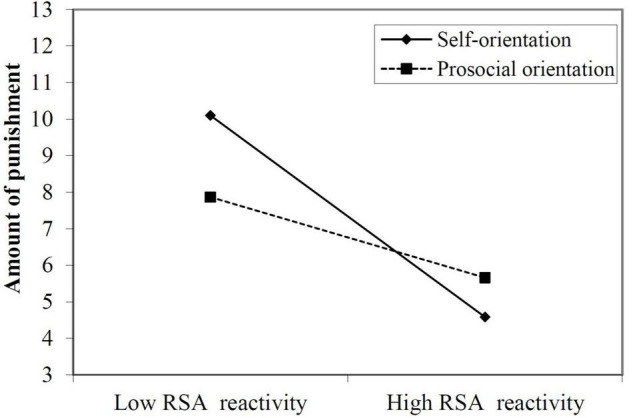
Simple slope plots of RSA reactivity × social value orientation on amount of punishment in third-party punishment task.

Finally, several linear regression analyses examined the interaction effect between RSA reactivity and social value orientation on prosocial behaviors. The results showed that the interaction effect of social value orientation and RSA reactivity on the frequency of punishment in the third-party task (β = 0.01, *t* = 0.07, *p* = 0.95) and the donated amount in the dictator game (β = 0.09, *t* = 0.90, *p* = 0.37) was not significant, but the interaction effect on the amount of punishment in the third-party task reached significance (Δ*R*^2^ = 0.06, β = 0.25, *t* = 2.23, *p* < 0.05). To further examine the interaction effect, we conducted simple slope analyses and found that participants with self-orientation reported less punishment in the third-party punishment task (β = −0.72, *t* = −3.72, *p* < 0.001; Figure 3) as RSA reactivity increased. In contrast, the amount of punishment of participants with prosocial orientation was not influenced by RSA reactivity (β = −0.19, *t* = −1.33, *p* > 0.05; Figure 3).

## Discussion

The findings of the current study revealed that acute psychological stress affected pure prosociality and prosocial third-party punishment, which were conceptualized as donations in the dictator game and the frequency and amount of punishment in the third-party punishment task. Moreover, these effects were found to vary as a function of participants’ gender and their social value orientations.

Specifically, consistent with the results of previous studies (von Dawans et al., 2011; Margittai et al., 2015; Potts et al., 2019), the results of the present study showed that compared with their counterparts under the resting control condition, those participants exposed to acute psychological stress were more generous in dictator games and tended to give the proposer more frequent and amounts punishment to maintain fairness in the third-party punishment task. These findings suggested that exposure to acute stress would lead to greater pure prosociality and increase participants’ more concern for the victims in the third-party punishment task, triggering more punishments to the norm violator, although they did not receive any overt benefits. These findings revealed the positive effect of acute stress on pure prosocial behaviors and provided more evidence to support the hypothesis of “tend and befriend,” in which individuals exhibit prosocial or affiliative behaviors following acute stress (Taylor et al., 2000; Taylor, 2006). Furthermore, the results of the current study revealed that there was a relationship between RSA reactivity and pure prosociality and prosocial third-party punishment. That is, those participants exhibited more donations in dictator games and more punishment behaviors in the third-party punishment task as their RSA reactivity to acute stress decreased. The findings suggested that RSA reactivity as an index reflecting the capacity of physiological response to a stressor might be a potential physical mechanism underlying individuals’ pure prosocial behaviors under acute stress.

One possible explanation is that although participants did not obtain gain from the receiver and even incurred the threat of reprisals, they still tended to engage in more prosocial behaviors as an adaptive coping strategy to alleviate negative emotions induced by acute stress and to further maintain mental health in the future (Raposa et al., 2016; Sollberger et al., 2016). Another potential explanation of the stress-induced increases in pure prosocial behaviors might be involved in the SIDI model of social decision-making (Yu, 2016). In the SIDI model, an intuition system processes information through an automatic, habitual, and evolutionarily based decision-making process, while a deliberation system processes information in a slow, goal-directed, and reasoning-based process. Stress altered the balance of the dual systems and facilitated a spontaneous and innate response in social situations. According to the social heuristic hypothesis, intuition drives people’s cooperation (Rand et al., 2014). Thus, stress increases the possibility of individuals engaging in pure prosocial behaviors in social situations.

Furthermore, the results of the current study showed that these effects of stress conditions or RSA reactivity on generosity in dictator games and the amount of punishment in the third-party punishment task did not vary between genders. These findings were consistent with the results of Zhen et al.’s (2021) study of the relationship between acute stress and third-party intervention and extended the original “tend-and-befriend” hypothesis that emphasized that women tend to protect themselves and their offspring by affiliating with others and engaging in prosocial behaviors after stress (Taylor et al., 2000). On the other hand, the results of the current study showed that there was still a gender effect of RSA reactivity on the frequency of punishment in the third-party punishment task. That is, male individuals exhibited a higher frequency of punishment than female individuals as RSA reactivity decreased. One possible explanation is that men and women have different perceptions of unfairness when other individuals behave unfairly, and males are more likely to punish norm-violating behaviors than females (Shang and Li, 2020). For example, Zheng et al. (2017) found that males rejected more unfair offers in the high- versus low-pressure context than females in an ultimatum game. Similarly, Rodriguez-Ruiz et al. (2019) found gender differences in the tendency to engage in Prosocial third-party punishment, with cooperating men being more likely to punish than cooperative women. This finding suggested that the way males and females engage in third-party punishment under acute stress could be different. Thus, these inconsistent findings also suggest that it is necessary for researchers to further examine these pure prosocial behaviors under acute stress in the future.

In addition, the results showed that social value orientation, in general, did not significantly moderate these effects of condition or RSA reactivity on prosocial behaviors. One possible explanation is that individuals’ prosocial decision-making depends on who they interact with. For example, Maeda and Hashimoto (2020) found that although individuals may be intuitive cooperators, they tended to act more cooperatively with their in-group members than with out-group members. However, on the other hand, the results showed that the impact of RSA reactivity on the amount of third-party punishment was still influenced by social value orientation (i.e., prosocial or self-orientation). Specifically, individuals with self-orientation exhibited more prosocial third-party punishment as RSA reactivity decreased, while RSA reactivity in individuals with prosocial orientation was not significantly related to their amount of prosocial third-party punishment. These findings suggested that compared with punishment frequency, the amount of prosocial third-party punishment was more sensitive to RSA reactivity in individuals with different social value orientations. One possible explanation is that individuals with a prosocial orientation were generally more sensitive to the feelings of others and thus were more inclined to engage in prosocial behaviors to maintain social relationships despite their RSA reactivity to stress. In contrast, with the change from low to high RSA reactivity, individuals with self-orientation under acute stress can dynamically distinguish themselves from other related representations and decreased egocentricity, leading to more empathetic concerns and prosocial behaviors (Tomova et al., 2014).

Some limitations of the current study should be noted. First, the sample of the current study was composed of participants of Chinese culture. Thus, the findings should be replicated in groups from other cultures. Second, previous studies found that while non-social, physical stress paradigms (e.g., the cold press task) are mainly involved in engagement of the SAM axis, which rapidly and quickly returns to baseline, the TSST used in most studies is characterized as a social evaluation mechanism and requires limbic engagement in threat appraisal to trigger HPA activation (exhibits a slower rise, peaking at 21–40 min; Schwabe et al., 2008). Thus, in the current study, it is possible that the stress effects on prosocial behaviors might have been attenuated, although prosocial decision-making was measured directly by the end of the TSST. Further studies are therefore necessary to examine the effect of stress-to-task latency more systematically on prosocial behaviors. In addition, in the current study, a pure resting control condition instead of the placebo-TSST was used as a control group which would influence the effect of condition or RSA reactivity on prosocial behaviors. Thus, it should be more cautious when explaining the results of the current study. Finally, studies have shown that several individual characteristics, such as personality, have potential effects on an acute stress on prosocial behaviors (Yu, 2016). Thus, future studies should consider the role of these variables in the relationships.

## Conclusion

The findings of the current study reveal more frequent and amount of prosocial third-party punishment and pure prosociality in dictator games after exposure to acute stress and provide further evidence for the “tend-and-befriend” hypothesis (Taylor et al., 2000; Taylor, 2006). More importantly, while social value orientation and gender in general did not significantly moderate these effects of condition or RSA reactivity on prosocial behaviors, the impacts of RSA reactivity to acute stress on the frequency and amount of prosocial third-party punishment were influenced by social value orientation and gender. Thus, the findings of the current study reveal two important factors of moderating in the relationship between acute psychological stress and pure prosocial behaviors and provide novel insights into the underlying physical mechanisms.

## Data Availability Statement

The datasets of the current study will not be available publicly because we do not have permissions from the participants to share the data. Requests to access the datasets should be directed to the corresponding author (LY), ying22690@sina.com.

## Ethics Statement

The studies involving human participants were reviewed and approved by the Ethics Committee of Zhejiang Sci-Tech University. The patients/participants provided their written informed consent to participate in this study.

## Author Contributions

LY and QY performed the study design, data analysis, interpretation, and drafted the manuscript. QY, XS, and CZ performed the data collection. LY provided critical revisions. All authors approved the final version of the manuscript for submission.

## Conflict of Interest

The authors declare that the research was conducted in the absence of any commercial or financial relationships that could be construed as a potential conflict of interest.

## Publisher’s Note

All claims expressed in this article are solely those of the authors and do not necessarily represent those of their affiliated organizations, or those of the publisher, the editors and the reviewers. Any product that may be evaluated in this article, or claim that may be made by its manufacturer, is not guaranteed or endorsed by the publisher.
